# Mediating effects of emotion regulation between socio-cognitive mindfulness and empathy in nurses: a cross-sectional study

**DOI:** 10.1186/s12912-022-01081-z

**Published:** 2022-11-09

**Authors:** Mikyoung Lee, Hyunyoung Park

**Affiliations:** 1 Department of Nursing, Kwangju Women’s University, 40 Gwangjuyeodai-gil, Gwangsan-gu, 62396 Gwangju, South Korea; 2 College of Nursing, Chonnam National University, 160 Baekseo-ro, Dong-Gu, 61469 Gwangju, South Korea

**Keywords:** Socio-cognitive mindfulness, Empathy, Emotion regulation, Reappraisal, Suppression

## Abstract

**Background:**

Acknowledging the under-examined research of socio-cognitive mindfulness and Gross’s emotion regulation strategies in nursing, this study investigated the relationships between socio-cognitive mindfulness, emotion regulation (i.e., reappraisal and suppression), and empathy among nurses. It also explored the mediating effects of emotion regulation.

**Methods:**

A cross-sectional quantitative study was conducted in a sample of 245 nurses from two university hospitals in South Korea. Convenience sampling was used to collect data between August 3 and September 29, 2020. Participants completed the questionnaire measuring their socio-cognitive mindfulness, emotion regulation, and empathy. Structural equation modeling and path analysis were conducted for data analysis.

**Results:**

Socio-cognitive mindfulness positively influenced emotion regulation of reappraisal (β = 0.404, *p* < 0.01) and empathy (β = 0.402, *p* < 0.01), but negatively influenced emotion regulation of suppression (β = −0.149, *p* < 0.05). Reappraisal positively influenced empathy (β = 0.341, *p* < 0.01), whereas suppression negatively influenced empathy (β = −0.127, *p* < 0.05). Importantly, emotion regulation of reappraisal mediated the association between socio-cognitive mindfulness and empathy (a X b = 0.107, *p* < 0.01).

**Conclusion:**

The findings indicate that socio-cognitive mindfulness is effective in improving empathy among nurses by enhancing reappraisal. This study can provide a foundation for developing socio-cognitive mindfulness or emotion regulation programs to improve empathy among nurses, which would ultimately lead to better nursing performance by increasing patient satisfaction.

## Background

As the fourth industrial revolution era accelerates, socio-emotional skills are becoming more essential for human beings to possess to counteract the influence of artificial intelligence [1]. As one of the main socio-emotional skills, empathy has been mentioned [2]. Empathy is defined as the ability to recognize others’ emotions and react to them appropriately by feeling an equivalent emotion in the self [3, 4]. Empathy is a necessary skill for human connectedness; in particular, a nurse’s empathy is a professional nursing skill based on a patient’s perspective [5]. It is described as wisdom, caring ability, communication and relationship, careful attention, self-awareness, and self-development [5]. A nurse’s empathy heavily influences the quality of the nurse-patient relationship and ultimately patient outcomes [6]. Patients who feel more empathy in their relationship with a nurse will likely perceive that they received good care. They consider a nurse who is trustworthy and exhibits professionalism based on empathy to be a good nurse; therefore, a nurse’s empathy becomes an important criterion for determining patient satisfaction [5, 7].

Interest in mindfulness has increased extensively in the field of healthcare over the last two decades due to its considerable benefits for healthcare providers [8]. Originally, mindfulness was taught as a strategy to reduce human suffering and cultivate empathy [9], which is particularly relevant in the nursing field. This mindfulness has been studied in two major theories: meditative mindfulness by Kabat-Zinn [10, 11] and socio-cognitive mindfulness by Langer [12]. Kabat-Zinn [10] defines meditative mindfulness as awareness by paying attention to the present moment purposefully and nonjudgmentally, while Langer [12] describes socio-cognitive mindfulness as a flexible status of mind focusing on the present moment by paying attention to context with novel ideas. As such, both mindfulness frameworks share the common feature of focused attention in the present moment. However, they operate as distinct processes in that meditative mindfulness involves a nonjudgmental awareness while socio-cognitive mindfulness involves cognitive processes [13].

Numerous studies on meditative mindfulness have identified the effectiveness of meditative mindfulness practice in the nursing field. For example, meditative mindfulness was negatively associated with nurses’ emotional exhaustion [14–17] but positively associated with physical and psychological well-being [14, 16], empathy [14, 18], personal accomplishment [15, 19, 20], and life satisfaction [17, 20]. For nursing students, mindfulness intervention was effective in alleviating stress [21–23], depression [22, 23], and anxiety [21–23]. In addition, a higher level of mindfulness was linked to enhancing nursing students’ sense of well-being [21], a reappraisal emotion strategy [24], empathy [21, 25, 26], self-esteem [27], and academic outcomes [28].

However, studies on socio-cognitive mindfulness are sparse in the nursing field despite its potential benefits. Nurses would benefit from applying socio-cognitive mindfulness to their nursing practice, given that socio-cognitive mindfulness helps individuals observe phenomena from various perspectives by considering contextual situations rather than fixed viewpoints [29] and enhances cognitive flexibility and intuitive insight by focusing attention on the present moment [30]. Limited previous studies on socio-cognitive mindfulness in nursing have found nurses’ socio-cognitive mindfulness to be positively related to personal accomplishment but negatively related to emotional exhaustion and depersonalization [31]. For nursing students, their socio-cognitive mindfulness positively influenced communication self-efficacy and empathy [32]. Nursing students’ socio-cognitive mindfulness had a positive influence on positive achievement emotions but a negative influence on negative achievement emotions [28, 33, 34]. In addition, their socio-cognitive mindfulness correlated positively with grit [34] and academic outcomes [28]. It was also positively related to a reappraisal emotion regulation strategy but negatively related to a suppression strategy regarding emotion regulation [33].

Regarding the literature on emotion regulation in nursing, earlier studies mainly focused on Hochschild’s [35] emotional labor with surface acting and deep acting [36–38], whereas Gross’s [39, 40] emotion regulation with reappraisal and suppression received little attention. Emotion regulation refers to “the processes by which individuals influence which emotions they have, when they have them, and how they experience and express these emotions” [39, p. 275]. It also refers to the competence that individuals can manage their emotional experiences and expressions [40]. Gross [39, 40] introduces two main strategies of emotion regulation: reappraisal and suppression. Reappraisal is a process of cognitive transformation that reinterprets emotional events to adjust for emotional influence, while suppression is a form of response modification to inhibit emotional expressions by actively suppressing them. Since reappraisal is an antecedent-oriented strategy that occurs early in the emotion-evoking process, it can effectively change the entire subsequent emotional process before an emotional response is fully produced [41]. Thus, reappraisal can be an effective way to reduce negative emotions or increase positive emotions. In comparison, suppression is a response-oriented strategy that occurs late in the emotional development process. Although it can reduce visible behaviors, it does not reduce the intensity of negative emotions but rather consumes cognitive resources by ruining memories of the information presented in the process of emotion regulation [41].

The use of a reappraisal strategy was associated with high well-being and interpersonal functioning (e.g., having better relationships and social support), whereas the use of a suppression strategy was associated with reverse consequences [41]. Furthermore, people who employed reappraisal reported a higher level of empathy compared to those who employed suppression [42–44]. Some studies also found that reappraisal was positively related to empathy, but suppression was negatively related to empathy [45–47]. Limited studies with nurses supported the benefits of applying reappraisal and the disadvantages of applying suppression. Specifically, nurses’ reappraisal strategy was positively related to positive emotions such as enjoyment and pride, but negatively related to negative emotions such as anxiety, anger, and frustration as well as emotional exhaustion [48]. In addition, the higher nurses’ emotional regulation ability, the higher their ability to empathize with others; that is, by regulating their own emotions, nurses’ ability to understand patients and coworkers increases through a mutual emotional exchange [49].

To date, the relationships between mindfulness, emotion regulation, and empathy in nursing have been examined in the meditative mindfulness construct [14, 18, 21, 24–26]. However, research on socio-cognitive mindfulness and its relationships with emotion regulation and empathy is deficient in the nursing field. Therefore, this study aimed to examine the relationships between socio-cognitive mindfulness, emotion regulation, and empathy among nurses as well as the mediating effects of emotion regulation in the link between socio-cognitive mindfulness and empathy. Grounded on previous research, we established the following research questions and hypotheses:

### Research question 1.

What are the structural relationships between socio-cognitive mindfulness, emotion regulation, and empathy among nurses?

Hypothesis 1: Socio-cognitive mindfulness is positively related to reappraisal but negatively related to suppression.

Hypothesis 2: Socio-cognitive mindfulness is positively related to empathy.

Hypothesis 3: Reappraisal is positively related to empathy, but suppression is negatively related to empathy.

### Research question 2.

Do emotion regulation strategies mediate the relationship between socio-cognitive mindfulness and empathy among nurses?

Hypothesis 4: Emotion regulation strategies (i.e., reappraisal and suppression) mediate the relationship between socio-cognitive mindfulness and empathy.

## Methods

### Research design

A cross-sectional quantitative study was conducted to examine the relationships between socio-cognitive mindfulness, emotion regulation, and empathy among nurses as well as the mediating effects of emotion regulation. The present research was reported according to the Strengthening the Reporting of Observational Studies in Epidemiology (STROBE) checklist for cross-sectional studies [50].

### Participants and procedure

A total of 245 clinical nurses consisting of 238 females (97.1%) and 7 males (2.9%) participated in the study. This sample size was suitable to perform structural equation modeling (SEM) for analysis in the present study, based on the recommendation by Kline [51] that a sample size over 200 is desirable. The participants were recruited from two university hospitals located in a metropolitan city in South Korea. The inclusion criteria were full-time nurses who worked for more than one year, understood the study purpose, and were willing to participate in the study. The exclusion criteria were nurses who worked less than one year or who refused to participate in the study. The participants’ ages ranged from 23 to 56 years, with a mean of 30.21 (SD = 6.85). The majority of participants (N = 192, 78.4%) had a bachelor’s degree, and 38 nurses (15.5%) had a master’s or doctoral degree. The length of working experience ranged from 1 to 33 years, with a mean of 7.12 years (SD = 6.92).

We used convenience sampling to collect data between August 3 and September 29, 2020. We visited the nursing directors in the two participating hospitals and explained the aims and significance of the study. Due to the COVID-19 pandemic situation in Korea, researchers were restricted from visiting nurses in units. Thus, we delivered the questionnaires to the nursing directors, and they distributed them to the head nurses in each unit. The head nurses distributed the questionnaires to nurses in their unit and collected the completed questionnaires. The questionnaires included a letter introducing the study, explaining the research purpose and procedure, as well as the measures of socio-cognitive mindfulness, emotion regulation, and empathy. Demographic questions were also included at the end of the questionnaire. The participants voluntarily filled in the consent form and completed the questionnaires within 15 min. A small gift of a pair of socks was provided as a reward. After collecting the answered questionnaires, the head nurses gave them to the hospital nursing director. In total, 260 questionnaires were distributed, and 250 (96.2%) were returned. After excluding five insincerely answered questionnaires, 245 were finally used for analysis.

### Measures

The instruments consisted of three measures to assess socio-cognitive mindfulness, emotion regulation strategies, and empathy among the participating nurses.

#### Socio-cognitive mindfulness

To measure the level of socio-cognitive mindfulness, we used the Korean validated Langer Mindfulness Scale (LMS) by Kim [52]. The LMS was originally developed by Bodner and Langer [53]. It consists of four dimensions of socio-cognitive mindfulness and includes 21 items: novelty seeking (six items), novelty producing (six items), flexibility (four items), and engagement (five items). Sample items are “I like to figure out how things work” for novelty seeking, “I make many novel contributions” for novelty producing, “I can behave in many different ways for a given situation” for flexibility, and “I get involved in almost everything I do” for engagement. The participants answered all of the items based on a 5-point Likert scale (1 = strongly disagree to 5 = strongly agree). We used the total score of the LMS for analysis, since we wanted to examine the associations between the main concepts of socio-cognitive mindfulness, emotion regulation, and empathy. In fact, the use of the total score for mindfulness would be more effective in maintaining a parsimonious model in the process of analysis [54]. The total score ranged from 21 to 105. The mean score was used, with a higher score indicating a higher level of socio-cognitive mindfulness. Cronbach’s alpha of the original scale was 0.89 for the total scale, and Cronbach’s alphas of the subscales, novelty seeking, novelty producing, flexibility, and engagement, were 0.74, 0.83, 0.54, and 0.63, respectively [53]. In this study, Cronbach’s alpha coefficient was 0.89 for the total scale. For the dimensions of novelty seeking, novelty producing, flexibility, and engagement, Cronbach’s alpha coefficients were acceptable at 0.72, 0.83, 0.62, and 0.70, respectively.

#### Emotion regulation

To assess the participants’ emotion regulation strategies, the Emotion Regulation Questionnaire (ERQ) developed by Gross and John [41] was adopted. For the present participants, we used the Korean validated ERQ by Shon [55], which was also used among Korean nurses with good reliabilities [48]. This scale is comprised of two dimensions: reappraisal and suppression. There are ten items in total: four reappraisal and six suppression items. Sample items are “I control my emotions by changing the way I think about the situation I’m in” for reappraisal, and “I control my emotions by not expressing them” for suppression. The participants responded on a 5-point Likert scale (1 = strongly disagree to 5 = strongly agree). The total score ranged from 10 to 50 and the mean score was utilized, with a higher score presenting a higher level of emotion regulation strategies. Cronbach’s alpha of the original scale was 0.82 for reappraisal and 0.76 for suppression [41]. In this study, the internal consistency of the total scale was good with a Cronbach’s alpha coefficient of 0.80. For the two dimensions, Cronbach’s alpha coefficients were 0.83 for reappraisal and 0.72 for suppression.

#### Empathy

To measure the participants’ empathy, we utilized the Korean validated empathy scale developed by Lee [56]. This scale was particularly developed and validated for nurses in Korea, demonstrating good reliability. This scale contains the three dimensions of communication, sensitivity, and insight. There are 13 items in total, with seven communication items (e.g., “I am aware of how to communicate with patients to encourage them”), three sensitivity items (e.g., “I am careful in my speech and behaviors so as to avoid hurting my patient’s feelings”), and three insight items (e.g., “I offer customized care to patients by taking their characteristics into consideration”). Responses to all of the items were rated on a 5-point Likert scale (1 = strongly disagree to 5 = strongly agree). The total score ranged from 17 to 85 and the mean score was used, with a higher score reflecting a higher level of empathy. Cronbach’s alpha of the original scale was 0.93 for the total scale, and the Cronbach’s alphas of the subscales, communication, sensitivity, and insight, were 0.89, 0.77, and 0.78, respectively [56]. In this study, the Cronbach’s alpha coefficients were 0.88 for the total scale, and 0.85, 0.72, and 0.69, respectively, for the dimensions of communication, sensitivity, and insight.

### Data Analysis

As an initial step to estimate means, standard deviations, and correlations for the study variables, we analyzed data using the SPSS 28 software program. Next, to investigate the relationships between the main constructs of socio-cognitive mindfulness, emotion regulation, and empathy, we performed SEM using the Mplus 8 program [57]. We chose Mplus since it offers a model fit index evaluating whether the model fits the data, corrects measurement error, and takes the full information maximum likelihood (FIML) approach in order to handle missing data [57]. We tested how well the model would fit the data, according to the recommended criteria of fit index: the comparative fit index (CFI) and the Tucker-Lewis Index (TLI) > 0.90 [51, 58], as well as the root-mean-square-error of approximation (RMSEA) and the standardized root-mean-square-residual (SRMR) < 0.08 [51, 59]. Finally, we conducted path analysis with Mplus 8 to discover the mediating effects of emotion regulation strategies in the association between socio-cognitive mindfulness and empathy.

## Results

### Preliminary results

Table 1 shows the means and standard deviations for the study variables as well as the results of the correlation analysis between the variables. The means of all variables were above the midpoint of the scales (2.5), with empathy the highest among them (M = 3.61, SD = 0.39). The mean level of socio-cognitive mindfulness was 3.21 (SD = 0.41). Regarding the emotion regulation strategies, participants responded higher to reappraisal (M = 3.33, SD = 0.56) than to suppression (M = 2.79, SD = 0.64).

Correlation analysis revealed that socio-cognitive mindfulness was positively related to the reappraisal strategy (r = 0.375, *p* < 0.01) and empathy (r = 0.584, *p* < 0.01). Participants’ reappraisal strategy was also positively related to the suppression strategy (r = 0.278, *p* < 0.01) and empathy (r = 0.444, *p* < 0.01). On the other hand, the suppression strategy was negatively related to socio-cognitive mindfulness (r = − 0.147, *p* < 0.05) and empathy (r = − 0.125, *p* < 0.05).

**Table 1:** Correlations, means, and standard deviations for the study variables (N = 245)

Variables	1	2	3	4
1. Socio-cognitive mindfulness	1			
2. Reappraisal	0.375**	1		
3. Suppression	−0.147*	0.278**	1	
4. Empathy	0.584**	0.444**	−0.125*	1
Mean ^a^	3.21	3.33	2.79	3.61
SD	0.41	0.56	0.64	0.39

Note. SD = Standard deviation. ^*a*^ Possible range 1 − 5. * *p* < 0.05. ** *p* < 0.01

### Relationships between socio-cognitive mindfulness, emotion regulation, and empathy (hypotheses 1–3)

**Fig. 1:**
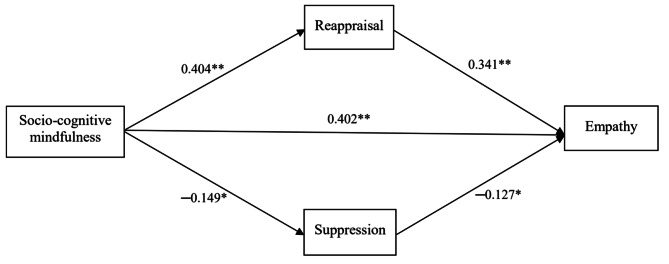
Structural equation model displaying parameter estimates for effects of socio-cognitive mindfulness on emotion regulation and empathy. * p < 0.05. ** p < 0.01

We conducted SEM with maximum likelihood using Mplus 8 [57] to analyze the path correlations between socio-cognitive mindfulness, emotion regulation, and empathy among nurses. Figure 1 presents the path coefficients or parameter estimates for the effects of socio-cognitive mindfulness on emotion regulation and empathy. This model was saturated, displaying CFI = 1.000, TLI = 1.000, RMSEA = 0.000, and SRMR = 0.000. In other words, the model fit indicates that the data fits the present model perfectly.

First, Hypothesis 1 was supported, reporting that socio-cognitive mindfulness had a positive influence on reappraisal (β = 0.404, *p* < 0.01) but a negative influence on suppression (β = −0.149, *p* < 0.05). Second, socio-cognitive mindfulness also had a positive effect on empathy (β = 0.402, *p* < 0.01), supporting Hypothesis 2. Finally, regarding the relationships between the emotion regulation strategies and empathy, reappraisal had a positive influence on empathy (β = 0.341, *p* < 0.01), but suppression had a negative influence on empathy (β = −0.127, *p* < 0.05) as expected (Hypothesis 3).

### Mediating effects of emotion regulation (hypothesis 4)

We implemented path analysis with Mplus 8 to explore whether nurses’ emotion regulation would mediate the relationships between their socio-cognitive mindfulness and empathy. Following Shrout and Bolger’s [60] suggestion, we included bootstrapping analysis with 5,000 times resampling for better power to achieve both direct and indirect effects with statistical significance. Path analysis uncovered that only the reappraisal strategy mediated the association between socio-cognitive mindfulness and empathy, partially supporting Hypothesis 4. Table 2 summarizes the total effect, direct effect, and indirect effect (i.e., mediating effect).

First, in the socio-cognitive mindfulness, reappraisal, and empathy link, socio-cognitive mindfulness had a positive influence on reappraisal (a = 0.366, *p* < 0.01), and reappraisal had a positive influence on empathy (b = 0.293, *p* < 0.01). The direct effect of socio-cognitive mindfulness on empathy decreased after controlling for the effect of reappraisal (c´= 0.436, *p* < 0.01), compared with the total effect (c = 0.543, *p* < 0.01). The indirect pathway or mediating effect through reappraisal was significant (a X b = 0.107, *p* < 0.01). This implies that reappraisal of the emotion regulation strategies partially mediates the relationship between socio-cognitive mindfulness and empathy among nurses.

Second, in the socio-cognitive mindfulness, suppression, and empathy link, socio-cognitive mindfulness had a negative influence on suppression (a = − 0.150, *p* < 0.05), and suppression had a negative influence on empathy (b = − 0.123, *p* < 0.05). The direct effect of socio-cognitive mindfulness on empathy decreased after controlling for the effect of reappraisal (c´= 0.523, *p* < 0.01), compared with the total effect (c = 0.542, *p* < 0.01). However, the indirect pathway through suppression was not significant (a X b = 0.019, ns), indicating no mediating role of suppression in the association between socio-cognitive mindfulness and empathy.

**Table 2:** Emotion regulation as a mediator of effects of socio-cognitive mindfulness on empathy (N = 245)

IV	M	DV			Total effect	Direct effect	Indirect effect
			IV→M (a)	M→DV (b)	IV→DV (c)	IV→DV (c´)	IV→M→DV (a X b)
SCMF	reappraisal	empathy	0.366**	0.293**	0.543**	0.436**	0.107**
SCMF	suppression	empathy	*−*0.150*	*−*0.123*	0.542**	0.523**	0.019

Note. IV = Independent variable; M = Mediator; DV = Dependent variable; SCMF = Socio-cognitive mindfulness. Standardized coefficients are reported. * *p* < 0.05. ** *p* < 0.01

## Discussion

This study investigated the relationships between socio-cognitive mindfulness, emotion regulation (i.e., reappraisal and suppression), and empathy among nurses as well as the mediating effects of emotion regulation in the link between socio-cognitive mindfulness and empathy. We found that nurses’ socio-cognitive mindfulness positively influenced reappraisal and empathy but negatively influenced suppression, and that reappraisal positively influenced empathy whereas suppression negatively influenced empathy. Importantly, emotion regulation of reappraisal mediated the association between socio-cognitive mindfulness and empathy.

First, regarding the relationship between socio-cognitive mindfulness and emotion regulation, the participants with higher socio-cognitive mindfulness used more reappraisal and less suppression as hypothesized. This finding is consistent with previous research that demonstrated a positive relationship between college students’ socio-cognitive mindfulness and reappraisal [61] as well as decreased maladaptive emotion regulation strategies, such as rumination, through a brief intervention based on socio-cognitive mindfulness [62]. A recent study involving nursing students also revealed that socio-cognitive mindfulness was positively related to reappraisal but negatively related to suppression [33]. Our result reflected that the main features of socio-cognitive mindfulness were being open to new information, constructing new categories by demonstrating creativity, and considering the present context [63]. That is, socio-cognitive mindfulness allows people to consider situations and phenomena from a variety of perspectives rather than a fixed perspective; thus, they could promote reappraisal by changing thoughts in a desirable direction, while reducing suppression which suppresses or conceals the expressions of their emotional experiences [29].

Furthermore, socio-cognitive mindfulness enhances cognitive flexibility and intuitive insight by focusing on the present [30], probably leading to the application of a reappraisal strategy. As Langer [64] claimed, our result indicated that nurses with higher socio-cognitive mindfulness could examine and accept new properties and aspects of information from various perspectives. In this process, nurses can become equipped with the ability to convert the way they think even in adverse nursing situations by applying a reappraisal strategy. In comparison, it is understandable that this quality of socio-cognitive mindfulness might have a rather negative influence on the suppression strategy among nurses.

Second, we found that nurses with a higher level of socio-cognitive mindfulness showed a higher level of empathy, suggesting that mindfulness assessed by a socio-cognitive framework is linked to nurses’ empathy. This indicated that mindful attitudes in nursing situations could enhance nurses’ empathy. This finding is similar to a previous study with nursing students reporting that through socio-cognitive mindfulness nursing students improved positive attributes such as empathy, communication skills, and self-efficacy [32]. Our result is also consistent with Trent et al.’s [13] study, which discovered a positive correlation between socio-cognitive mindfulness and both affective and cognitive empathy in adults. Furthermore, recent definitions of mindfulness involve compassion, kindness, sympathetic joy, and openhearted behaviors with empathetic perspectives [65, 66]. This is reflected in our finding of a positive association between socio-cognitive mindfulness and empathy.

In addition, the positive relationship between socio-cognitive mindfulness and empathy is understandable, considering the similarities between both mindfulness frameworks. To illustrate, the core mechanism of both mindfulness frameworks includes attention and managing emotions, and both highlight awareness and focused attention on the present moment [13, 67]. In fact, some previous studies reported a positive correlation between meditative mindfulness and socio-cognitive mindfulness [13, 28, 68]. Positive correlations between meditative mindfulness and empathy have also been found in previous studies [69, 70], including those in the nursing field [14, 18, 21, 25, 26].

Third, regarding the associations between emotion regulation and empathy, reappraisal positively influenced empathy, but suppression negatively influenced empathy. This implied that the nurses who applied a reappraisal strategy possessed higher empathy than those who used a suppression strategy in nursing environments. This result is in line with studies that reported a higher level of empathy in individuals using a reappraisal strategy but a lower level of empathy in those using a suppression strategy [42–44]. Other studies also found a positive correlation between reappraisal and empathy as well as a negative correlation between suppression and empathy [45–47].

According to Gross and John [41], the use of reappraisal is associated with positive social consequences such as emotion sharing, perceived amiability, and keeping close relationships with others. This attribute of reappraisal may have generated a positive relationship between reappraisal and empathy among nurses in the present study. Furthermore, reappraisal is a cognitive strategy to modify emotional influences [39, 40], and empathy also involves cognitive factors such as perspective taking that allows people to understand and respond to the emotions felt by others [4, 42]. This may explain the positive relationship between reappraisal and empathy in the present study, reinforcing that nurses could improve their ability to empathize with patients and coworkers by effectively regulating their own emotions [49]. In contrast, the use of suppression is related to negative social effects such as decreased emotion sharing and reduced close relationships with others [41]. Thus, individuals using a suppression strategy may have a lower capacity to exhibit empathy, given that empathy skills require a certain degree of feeling socially connected to others [45]. The negative association between suppression and empathy may have arisen because when people employ a suppression strategy, they undergo incongruence between the emotions they are actually feeling and the ones they are expressing; this may impede social connectedness to others, possibly reducing the ability to empathize with others [71].

Finally, mediation analysis revealed that reappraisal of the emotion regulation strategies appeared to be a mediator of the effect of socio-cognitive mindfulness on empathy, demonstrating both the direct impact of socio-cognitive mindfulness and the indirect impact of reappraisal on empathy. That is, socio-cognitive mindfulness was indirectly associated with increased empathy through higher reappraisal, indicating that nurses with a higher level of socio-cognitive mindfulness may use more reappraisal, which in turn enables them to show higher empathy. Reappraisal was documented as a partial mediator, meaning that reappraisal was explaining only a part of the association between socio-cognitive mindfulness and empathy. There might be other variables to account for the link between socio-cognitive mindfulness and empathy. While future research should investigate this association in detail, the present mediating role of reappraisal supports that emotion regulation could be one explanation for the association between socio-cognitive mindfulness and empathy among nurses. No mediating role of suppression might be due to the positive correlation between reappraisal and suppression in this study, which has also been found in previous studies [72, 73]. Another reason could be that the cross-sectional design of the study might have restricted the comprehensive understanding of the associations between socio-cognitive mindfulness, emotion regulation, and empathy. In sum, the present mediating result emphasizes the relevance of a reappraisal strategy in the nursing context, further highlighting the need for training programs to improve reappraisal among nurses.

Despite these meaningful findings, we acknowledge some limitations in our study. First, we used cross-sectional data, and so the causal links between the study variables should be carefully interpreted. In particular, mediation analysis using cross-sectional and non-experimental data cannot exclude alternative models to investigate causal directions [69]. Future research might address this limitation by utilizing longitudinal and experimental designs to explore the association between the study variables as well as the mediating roles. Second, self-reported measures were used to evaluate study variables, which might have led to biased responses of the participants. Instead of expressing their authentic answers, the nurses participating in this study might have considered socially desirable answers that general people expect of a nurse, regarding the constructs of mindfulness, emotion regulation, and empathy. To compensate for this shortcoming, future research could employ intervention studies to identify whether training programs aimed at cultivating nurses’ socio-cognitive mindfulness and effective emotion regulation strategies could improve empathy in nurses. Third, the current sample consisted of nurses from two university hospitals located in South Korea. Thus, it is prudent to refrain from regarding this sample as representative of nurses and generalizing the results to other nurse populations. Future studies should include more varied nurse samples in terms of hospitals, gender, or countries to see if the findings are replicable and thus expanding on this research.

## Conclusion

By integrating the under-examined research of Langer’s [12] socio-cognitive mindfulness and Gross’s [39, 40] emotion regulation strategies in nursing, this study expands the literature on nurses’ mindfulness and emotion management. The study confirmed significant relationships between socio-cognitive mindfulness, emotion regulation, and empathy among nurses in Korea. Particularly, the present study is one of the first studies discovering the mediating role of emotion regulation in the association between socio-cognitive mindfulness and empathy in nurses. This finding highlights the relevance of emotion regulation to enhance empathy in the nursing context. Overall, the findings indicate that socio-cognitive mindfulness is effective in improving empathy among nurses by enhancing reappraisal. This study can provide a foundation for developing socio-cognitive mindfulness or emotion regulation programs to improve empathy among nurses, which would ultimately lead to better nursing performance by increasing patient satisfaction.

## Data Availability

The data presented in this study are available upon request from the corresponding author.

## Declarations

Permissions to collect data were also received from the nursing directors of the two participating hospitals. Informed consent was obtained from all participants, and the participants voluntarily completed the questionnaires. They were assured that their data would be strictly protected and used only for this research. They were also informed that they had the right to decline and withdraw from the study at any time if they wished.

## Abbreviations

SEM: Structural equation modeling
SD: Standard deviation
COVID-19: Corona Virus Disease-19
LMS: Langer mindfulness scale
ERQ: Emotion regulation questionnaire
CFI: Comparative fit index
TLI: Tucker-Lewis index
RMSEA: Root-mean-square-error of approximation
SRMR: Standardized root-mean-square-residual
IV: Independent variable
M: Mediator
DV: Dependent variable
SCMF: Socio-cognitive mindfulness

## References

[1] Lee SY (2017). Educational psychology in the age of the fourth industrial revolution. Korean Educ Rev.

[2] Lee M (2020). Relationships between achievement goals, emotions, and academic performance in nursing students. KALCI.

[3] Baron-Cohen S, Wheelwright S (2004). The empathy quotient: an investigation of adults with Asperger syndrome or high functioning autism, and normal sex differences. J Autism Dev Disord.

[4] Davis MH (1983). Measuring individual differences in empathy: Evidence for a multidimensional approach. J Pers Soc Psychol.

[5] Halldorsdottir S (2012). Nursing as compassionate competence: A theory on professional nursing care based on the patient’s perspective. Int J Hum Caring.

[6] Alligood MR, Chitty KK (2005). Nursing theory: The basis for professional nursing. Professional Nursing: Concepts and Challenges.

[7] Hill R (2014). Compassion, quality and standards of care. Nurse Prescribing.

[8] White L (2014). Mindfulness in nursing: an evolutionary concept analysis. J Adv Nurs.

[9] Ludwig DS, Kabat-Zinn J (2008). Mindfulness in medicine. JAMA.

[10] Kabat-Zinn J (1982). An outpatient program in behavioral medicine for chronic pain patients based on the practice of mindfulness meditation: Theoretical considerations and preliminary results. Gen Hosp Psychiatry.

[11] Kabat-Zinn J (2003). Mindfulness-based interventions in context: past, present, and future. Clin Psychol Sci Pract.

[12] Langer EJ. Mindfulness. Cambridge: De Capo Press;1989.

[13] Trent NL, Park C, Bercovitz K, Chapman IM (2016). Trait socio-cognitive mindfulness is related to affective and cognitive empathy. J Adult Dev.

[14] Bazarko D, Cate RA, Azocar F, Kreitzer MJ (2013). The impact of an innovative mindfulness-based stress reduction program on the health and well-being of nurses employed in a corporate setting. J Workplace Behav Health.

[15] Cohen-Katz J, Wiley SD, Capuano T, Baker DM, Kimmel S, Shapiro S (2005). The effects of mindfulness-based stress reduction on nurse burnout and stress: A quantitative and qualitative study, Part II. Holist Nurs Pract.

[16] Dos Santos TM, Kozasa EH, Carmagnani IS, Tanaka LH, Lacerda SS, Nogueira-Martins LA (2016). Positive effects of a stress reduction program based on mindfulness meditation in Brazilian nursing professionals: Qualitative and quantitative evaluation. Explore.

[17] Duarte J, Pinto-Gouveia J (2016). Effectiveness of a mindfulness-based intervention on oncology nurses’ burnout and compassion fatigue symptoms: A non-randomized study. Int J Nurs Stud.

[18] Pérez-Fuentes MDC, Gázquez Linares JJ, Molero Jurado MDM, Simón Márquez MDM, Martos Martínez Á (2020). The mediating role of cognitive and affective empathy in the relationship of mindfulness with engagement in nursing. BMC Public Health.

[19] Gauthier T, Meyer RML, Grefe D, Gold JI (2015). An on-the-job mindfulness-based intervention for pediatric ICU nurses: A pilot. J Pediatr Nurs.

[20] Mackenzie CS, Poulin PA, Seidman-Carlson R (2006). A brief mindfulness-based stress reduction intervention for nurses and nurse aides. Appl Nurs Res.

[21] Beddoe AE, Murphy SO (2004). Does mindfulness decrease stress and foster empathy among nursing students?. J Nurs Educ.

[22] Song Y, Lindquist R (2015). Effects of mindfulness-based stress reduction on depression, anxiety, stress and mindfulness in Korean nursing students. Nurse Educ Today.

[23] Spadaro KC, Hunker DF (2016). Exploring the effects of an online asynchronous mindfulness meditation intervention with nursing students on stress, mood, and cognition: A descriptive study. Nurse Educ Today.

[24] Dubert CJ, Schumacher AM, Locker L, Gutierrez AP, Barnes VA (2016). Mindfulness and emotion regulation among nursing students: Investigating the mediation effect of working memory capacity. Mindfulness.

[25] Ardenghi S, Russo S, Luciani M, Salvarani V, Rampoldi G, Bani M, Ausili D, Mauro S, Strepparava MG. The association between dispositional mindfulness and empathy among undergraduate nursing students: A multicenter cross-sectional study. Curr Psychol. 2022: 1–9. 10.1007/s12144-022-02829-1.

[26] Gür GC, Yilmaz E (2020). The effects of mindfulness-based empathy training on empathy and aged discrimination in nursing students: A randomised controlled trial. Complement Ther Clin Pract.

[27] Choi YS, Kim MA (2019). The effect of mindfulness-based cognitive therapy program on stress, self-esteem and depression of nursing students. J Korea Cont Assoc.

[28] Lee M, Jang KS (2021). Nursing students’ meditative and sociocognitive mindfulness, achievement emotions, and academic outcomes: Mediating effects of emotions. Nurse Educ.

[29] Carson SH, Langer EJ (2006). Mindfulness and self-acceptance. J Ration-Emot Cogn Behav Ther.

[30] Kahneman D (2011). Thinking, fast and slow.

[31] Heard PL. The relationship and effects of mindfulness on comfort, work satisfaction, and burnout among nurses who provide direct patient care. PhD thesis, University of Southern Mississippi, Hattiesburg, MS, USA, 2010.

[32] Sundling V, Sundler AJ, Holmström IK, Kristensen DV, Eide H (2017). Mindfulness predicts student nurses’ communication self-efficacy: A cross-national comparative study. Patient Educ Couns.

[33] Lee M, Jang KS (2021). Mediating effects of emotion regulation between socio-cognitive mindfulness and achievement emotions in nursing students. Healthcare.

[34] Lee M (2022). Nursing students’ grit, socio-cognitive mindfulness, and achievement emotions: mediating effects of socio-cognitive mindfulness. Int J Environ Res Public Health.

[35] Hochschild AR (1983). The managed heart: Commercialization of human feeling.

[36] Gray B (2009). The emotional labour of nursing: Defining and managing emotions in nursing work. Nurse Educ Today.

[37] Lee M, Jang KS (2019). Nurses’ emotions, emotional labor, and job satisfaction. Int J Workplace Health Manag.

[38] Mauno S, Ruokolainen M, Kinnunen U, De Bloom J (2016). Emotional labour and work engagement among nurses: Examining perceived compassion, leadership and work ethic as stress buffers. J Adv Nurs.

[39] Gross JJ (1998). Antecedent- and response-focused emotion regulation: divergent consequences for experience, expression and physiology. J Pers Soc Psychol.

[40] Gross JJ (2002). Emotion regulation: Affective, cognitive, and social consequences. Psychophysiology.

[41] Gross JJ, John OP (2003). Individual differences in two emotion regulation processes: Implications for affect, relationships, and well-being. J Pers Soc Psychol.

[42] Laghi F, Lonigro A, Pallini S, Baiocco R (2018). Emotion regulation and empathy: Which relation with social conduct?. J Genet Psychol.

[43] Troyer D, Greitemeyer T (2018). The impact of attachment orientations on empathy in adults: Considering the mediating role of emotion regulation strategies and negative affectivity. Pers Individ Differ.

[44] Witvliet CV, Hofelich Mohr AJ, Hinman NG, Knoll RW (2015). Transforming or restraining rumination: The impact of compassionate reappraisal versus emotion suppression on empathy, forgiveness, and affective psychophysiology. J Posit Psychol.

[45] Lebowitz MS, Dovidio J (2015). Implications of emotion regulation strategies for empathic concern, social attitudes, and helping behavior. Emotion.

[46] Lockwood PL, Seara-Cardoso A, Viding E (2014). Emotion regulation moderates the association between empathy and prosocial behavior. PLoS ONE.

[47] Powell PA (2018). Individual differences in emotion regulation moderate the associations between empathy and affective distress. Motiv Emot.

[48] Lee M, Jang KS (2019). Nurses’ emotions, emotion regulation and emotional exhaustion. Int J Organ Anal.

[49] Salvarani V, Rampoldi G, Ardenghi S, Bani M, Blasi P, Ausili D, Di Mauro S, Strepparava MG (2019). Protecting emergency room nurses from burnout: The role of dispositional mindfulness, emotion regulation and empathy. J Nurs Manag.

[50] Von Elm E, Altman DG, Egger M, Pocock SJ, Gøtzsche PC, Vandenbroucke JP (2014). The Strengthening the Reporting of Observational Studies in Epidemiology (STROBE) Statement: guidelines for reporting observational studies. Int J Surg.

[51] Kline RB (2015). Principles and practice of structural equation modeling.

[52] Kim BS (2010). Validation of the Korean version of the mindfulness/mindlessness scale. Korean J Couns.

[53] Bodner TE, Langer EJ. Individual differences in mindfulness: The mindfulness/mindlessness scale. In Proceedings of the 13th Annual American Psychological Society Convention, Toronto, ON, Canada, 14–17 June 2001.

[54] Pepping CA, O’Donovan A, Zimmer-Gembeck MJ, Hanisch M (2015). Individual differences in attachment and eating pathology: The mediating role of mindfulness. Pers Individ Differ.

[55] Shon JM. Individual differences in two regulation strategies: cognitive reappraiser vs. emotion suppressor. Master’s thesis, Seoul National University, Seoul, Korea, 2005.

[56] Lee YJ. Development of the compassionate competence scale for nurses. PhD thesis, Korea University, Seoul, Korea, 2014.

[57] Muthén LK, Muthén BO. Mplus user’s guide. 8th ed. Los Angeles: Muthén & Muthén; 1998–2017.

[58] Lance CE, Butts MM, Michels LC (2006). The sources of four commonly reported cutoff criteria: What did they really say?. Organ Res Methods.

[59] Browne MW, Cudeck R (1992). Alternative ways of assessing model fit. Sociol Methods Res.

[60] Shrout PE, Bolger N (2002). Mediation in experimental and nonexperimental studies: New procedures and recommendations. Psychol Methods.

[61] Haigh EA, Moore MT, Kashdan TB, Fresco DM (2011). Examination of the factor structure and concurrent validity of the Langer mindfulness/mindlessness scale. Assess.

[62] Huffziger S, Kuehner C (2009). Rumination, distraction, and mindful self-focus in depressed patients. Behav Res Ther.

[63] Langer EJ, Moldoveanu M (2000). The construct of mindfulness. J Soc Issues.

[64] Langer EJ (2007). On becoming an artist: Reinventing yourself through mindful creativity.

[65] Baer RA (2019). Assessment of mindfulness by self-report. Curr Opin Psychol.

[66] Voci A, Veneziani CA, Fuochi G (2019). Relating mindfulness, heartfulness, and psychological well-being: The role of self-compassion and gratitude. Mindfulness.

[67] Hart R, Ivtzan I, Hart D (2013). Mind the gap in mindfulness research: a comparative account of the leading schools of thought. Rev Gen Psychol.

[68] Lee SH. The effect of mothers’ mindfulness on adolescents’ behavior problems: The mediating effect of parenting attitudes and parent-child relationship satisfaction. Master’s thesis, Ewha Womans University, Seoul, Korea, 2017.

[69] Fuochi G, Voci A (2020). A deeper look at the relationship between dispositional mindfulness and empathy: Meditation experience as a moderator and dereification processes as mediators. Pers Individ Differ.

[70] Luberto CM, Shinday N, Song R, Philpotts LL, Park ER, Fricchione GL, Yeh GY (2018). A systematic review and meta-analysis of the effects of meditation on empathy, compassion, and prosocial behaviors. Mindfulness.

[71] English T, John OP (2013). Understanding the social effects of emotion regulation: the mediating role of authenticity for individual differences in suppression. Emotion.

[72] Lee M, Pekrun R, Taxer JL, Schutz PA, Vogl E, Xie X (2016). Teachers’ emotions and emotion management: Integrating emotion regulation theory with emotional labor research. Soc Psychol Educ.

[73] Matsumoto D, Yoo SH, Nakagawa S (2008). Culture, emotion regulation, and adjustment. J Pers Soc Psychol.

